# Infantile Convulsions, Etc.

**Published:** 1891-01

**Authors:** A. Jacobi

**Affiliations:** New York City


### INFANTILE CONVULSIONS.

BY DR. A. JACOBI, NEW YORK CITY.

Dr. Jacobi first orders a purgative dose of calomel and then follows in a few hours by:

℞ Chloral hydrat., gr. iv.

Potas. bromid., gr. viij.

Aquæ, } aa f ℨ j. M.

Syrupi, }

Sig. One dose for a child two years old.

